# Projected Dietary Intake of Zinc, Copper, and Cerium from Consumption of Carrot (*Daucus carota*) Exposed to Metal Oxide Nanoparticles or Metal Ions

**DOI:** 10.3389/fpls.2016.00188

**Published:** 2016-02-24

**Authors:** Stephen D. Ebbs, Scott J. Bradfield, Pawan Kumar, Jason C. White, Xingmao Ma

**Affiliations:** ^1^Department of Plant Biology and Center for Ecology, Southern Illinois University, CarbondaleIL, USA; ^2^Department of Analytical Chemistry, The Connecticut Agricultural Experiment Station, New HavenCT, USA; ^3^Zachry Department of Civil Engineering, Texas A&M University, College StationTX, USA

**Keywords:** nanoparticles, nanomaterials, metals, food safety, copper, zinc, ZnO, CuO, CeO_2_

## Abstract

The expanding production and use of engineered nanomaterials (ENMs) have raised concerns about the potential risk of those materials to food safety and human health. In a prior study, the accumulation of Zn, Cu, and Ce from ZnO, CuO, or CeO_2_, respectively, was examined in carrot (*Daucus carota* L.) grown in sand culture in comparison to accumulation from exposure to equivalent concentrations of ionic Zn^2+^, Cu^2+^, or Ce^4+^. The fresh weight concentration data for peeled and unpeeled carrots were used to project dietary intake of each metal by seven age-mass classes from child to adult based on consumption of a single serving of carrot. Dietary intake was compared to the oral reference dose (oral RfD) for chronic toxicity for Zn or Cu and estimated mean and median oral RfD values for Ce based on nine other rare earth elements. Reverse dietary intake calculations were also conducted to estimate the number of servings of carrot, the mass of carrot consumed, or the tissue concentration of Zn, Cu, or Ce that would cause the oral RfD to be exceeded upon consumption. The projections indicated for Zn and Cu, the oral RfD would be exceeded in only a few highly unrealistic scenarios of exceedingly high Zn or Cu concentrations in the substrate from ZnO or CuO or consumption of excessive amounts of unpeeled carrot. The implications associated with the presence of Ce in the carrot tissues depended upon whether the mean or median oral RfD value from the rare earth elements was used as a basis for comparison. The calculations further indicated that peeling carrots reduced the projected dietary intake by one to two orders of magnitude for both ENM- and ionic-treated carrots. Overall in terms of total metal concentration, the results suggested no specific impact of the ENM form on dietary intake. The effort here provided a conservative view of the potential dietary intake of these three metals that might result from consumption of carrots exposed to nanomaterials (NMs) and how peeling mitigated that dietary intake. The results also demonstrate the potential utility of dietary intake projections for examining potential risks of NM exposure from agricultural foods.

## Introduction

Globally there is a considerable emphasis on both the development of nanotechnology and the evaluation of the potential detrimental effects of nanomaterials (NMs) to living organisms. The field of nanotoxicology is rapidly maturing as an increasing number of studies are published that describe the toxicity of NMs (for reviews, see Grieger et al., 2011; Menard et al., 2011; Hristozov et al., 2012; Miralles et al., 2012; Pan and Xing, 2012; Bour et al., 2015). Such research has been driven by concerns about NM release into the environment and the commensurate effects on human and ecological health. The latter has received considerable attention in the literature, with a significant focus on the toxicity of NMs to microorganisms, plants, and aquatic organisms. Aside from the occupational context, ingestion through the diet represents one possible significant route of human exposure. This has prompted a growing number of questions about the impact of engineered nanomaterials (ENMs) on food safety.

For the metallic oxide ENMs, these materials present an added complexity in that they are not stable in environmental matrices and undergo dissolution to release the component metal ions. This has been shown to occur for ZnO, CuO, and CeO_2_ (Franklin et al., 2007; Dimkpa et al., 2012b, 2013; Tourinho et al., 2012; Notter et al., 2014; Schwabe et al., 2014, 2015; Odzak et al., 2015). Roots from food crops are thus exposed to mixtures of the parent ENM and the metal ion released from the ENM. In the absence of specific data to confirm whether intact ENMs in food present a specific risk, the alternate perspective has been to consider risk based on the accumulated metal irrespective of its chemical form. That is, consider the food safety risk based on the total concentration of Zn or Cu, for example, not the specific amount of ZnO or CuO. Until such time as there are limits for ENMs, this is a logical approach as there are specific guidelines in foods for a variety of metals. A valuable contrast to the potential impact of the ENMs is to make comparisons to that presented by the ionic form of the metal associated with the metallic oxide ENM. The purpose of such comparisons has been to assess whether exposure to the ENM resulted in toxicity equivalent to, less than, or greater than the metal ion itself. The same strategy can also be used in evaluating the impact that consumption of plants exposed the metallic oxide ENMs might have on humans.

There has been little effort to extrapolate from accumulation of metallic oxide ENMs in agricultural plants to the potential exposure through the diet to the metals associated with these materials. There are some reports for TiO_2_ from some processed foods and nutritional supplements (Fröhlich and Roblegg, 2012; Weir et al., 2012; Reed et al., 2014). However, there are no established limits for Ti in food to provide a framework to evaluate the implications. Perhaps the only relevant study to relate accumulation to potential risk involved soybean (*Glycine max*) and ZnO (Priester et al., 2012) where the authors related the Zn concentration in the edible tissues to the tolerable upper intake levels (UILs) of Zn in food established by the National Institute of Health (https://ods.od.nih.gov/factsheets/Zinc-HealthProfessional/). Their results, based on the projected mass of Zn ingested not the dietary intake or dose and compared to the UILs, suggested no potential toxicity of Zn to humans from consumption. Aside from this study, there is no other tangible data we are aware of that relates the accumulation of metals from metallic oxide ENMs in edible plant tissues to relevant guidelines or limits.

In a prior study, the accumulation of Zn, Cu, or Ce from ZnO, CuO, or CeO_2_, respectively, was examined in carrot (*Daucus carota* L.) in comparison to accumulation from exposure to equivalent concentrations of ionic Zn^2+^, Cu^2+^, or Ce^4+^ (Ebbs et al., 2016). Carrot was selected for that study because the vast majority of studies on the accumulation of metals from metallic oxide ENMs have demonstrated that the largest fraction of the metals is typically retained in the roots. This would imply that belowground root, tuberous, and bulb vegetables, due to their direct contact with the growth substrate, would likely accumulate higher concentrations of metals from metallic oxide ENMs than edible stems, leaves, fruits, or seeds. There were three clear trends evident in the results of that research. The first was that the outer surface peel (i.e., the periderm layer) of the carrot taproot (which is often removed during preparation of the vegetable) was where the majority of the metals from the ENM and ionic forms accumulated. However, the ionic metal passed through this outer layer and accumulated at higher concentrations in the edible taproot flesh than the corresponding ENM treatments. Finally overall, more of the ionic metal accumulated in all the plant tissues than metal from the ENMs, suggesting that the ENMs were less of an accumulation risk than free metal ions. However, that study did not relate the accumulation or spatial distribution of metals in the carrot taproot to any possible risks for human consumption.

For the study described here, the metal accumulation data from this prior study was used to estimate the dietary intake that would occur from consumption of either fresh peeled or fresh unpeeled carrot grown under each of the exposure regimes. Dietary intake was calculated for specific age-mass classes from child to adult and was intended to provide a comparison of projected intake between the ENM and ionic forms. The estimated dietary intakes from the ENM and ionic treatments were compared to an established or estimated oral reference dose (oral RfD) for chronic exposure as a relevant endpoint. The oral RfD refers to the daily exposure of the human population to a potential hazard that is likely to be without risk of deleterious effects during a lifetime (Agency for Toxic Substances and Disease Registry [ATSDR], 2004). An additional objective of the dietary projections here was to further illustrate the implications of the retention of metals in the taproot peel to the potential dietary intake of the metal. As some do not peel carrots prior to consumption, the intent was to illustrate the degree to which a simple preparation step like peeling might alter the potential intake of metals from the two sources (ENM or metal ion). The overarching goal of this research was to provide data that would contribute to ongoing risk assessments of the potential detrimental impacts of metallic oxide ENMs to food safety.

## Materials and Methods

### Forward Projections of Dietary Intake of Zn, Cu, or Ce

The forward projections of dietary intake utilized the fresh weight tissue concentration of Zn, Cu, or Ce in the treated carrots (*Daucus carota* cv. Danvers Half Long) to estimate the projected dietary intake resulting from human consumption of those treated vegetables. Complete details on the methodology used to produce the carrot plants are provided elsewhere (Ebbs et al., 2016) and are summarized in the Supplementary Material. Human dietary intake was calculated using the following formula, where dietary intake is expressed in units of μg kg^-1^ d^-1^, tissue concentration is expressed in mg kg FW^-1^, mass consumed and body mass are both in kg, and a value of 1,000 was used to convert milligrams to micrograms (Ebbs et al., 2006, 2010).

$$\mathrm{Dietary}\;\mathrm{intake}=\frac{\mathrm{tissue}\;\mathrm{concentration}\times \mathrm{mass}\;\mathrm{per}\;\mathrm{serving}\times \#\mathrm{of}\;\mathrm{servings}\times 1,000}{\mathrm{body}\;\mathrm{mass}}$$

Dietary intake was calculated for consumption of the carrot taproot exposed either to the ENM or the corresponding ions and for either peeled or unpeeled carrots. For peeled carrots, the fresh weight tissue concentration of Zn, Cu, or Ce was calculated using the concentration obtained from the analysis of the dried tissues (i.e., dry weight concentration) and percent water content of the fresh tissues. To derive the metal concentration in the fresh, unpeeled carrots, the dry weight concentration in the peels and the peeled taproot for each harvested carrot was determined. Using the dry weight biomass for each tissue (i.e., peel or taproot), the total dry weight mass of each element in the peels or peeled storage organ for each harvested carrot was then determined. As the fresh weight mass for each peeled carrot taproot and its peels were collected at harvest for each individual carrot taproot, it was possible to then calculate the total fresh weight concentration of each metal in the unpeeled carrot. The dietary intake (μg kg^-1^ d^-1^) was then calculated using these fresh weight concentrations for either peeled or unpeeled carrots, the typical mass of raw carrot tissue consumed per serving (0.062 g FW; Pennington and Douglass, 1994), and standard body mass values for age groups from 1 to 3 years through adult females and males (National Academy of Sciences, 2001). The specific age-mass classes used were child 1–3 years (13 kg), child 4–8 years (22 kg), children 9–13 years (40 kg), adolescent female 14–18 years (57 kg), adolescent male 14–18 years (64 kg), adult female 19+ years (61 kg), and adult male 19+ years (76 kg); thus creating seven age-mass classes. To provide a frame of reference for the interpreting the calculated dietary intake rates, the values for Zn and Cu were compared to the chronic oral RfD for those elements. The values used for Zn (300 μg kg^-1^ d^-1^) and Cu (40 μg kg^-1^ d^-1^) were obtained from the U.S. EPA Health Effects Assessment Summary Tables (HEAST; USEPA, 1997). There is no established oral RfD for Ce. To provide a surrogate parameter for comparison, the oral RfD values for nine other rare earth elements (Eu, La, Ly, Nd, Pr, Sc, Sm, U, Y) were obtained (USEPA, 2012). As the oral RfD values for these elements range from 0.9 to 500 μg kg^-1^ d^-1^, it was not possible to select the value for a single element to use in this comparison. Instead, the mean (171.9 mg kg^-1^ d^-1^) and median (5 μg kg^-1^ d^-1^) values were calculated for these elements and both were used here as indicators of the oral RfD for Ce.

### Reverse Projections of Dietary Intake of Zn, Cu, or Ce

The reverse projections conducted here used the dietary intake equation above and the oral RfD values for Zn, Cu, or Ce to solve for individual parameters (e.g., number of servings, fresh weight mass per serving, or fresh weight tissue concentration). The equations were solved separately for each age-mass class.

## Results

### Forward Projections of Dietary Intake of Zn, Cu, or Ce

**FIGURE 1:**
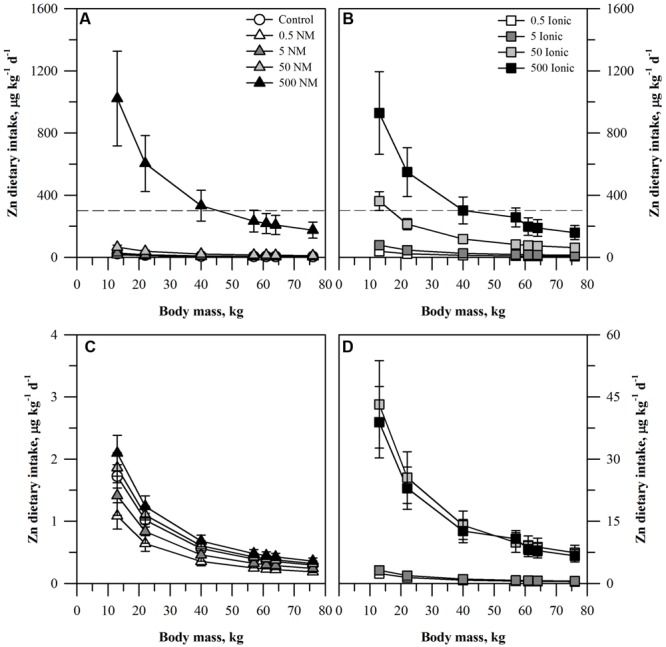
**Dietary intake models for Zn based on consumption of a single serving of unpeeled **(A,B)** or peeled carrot **(C,D)** grown in sand culture in the presence of four final concentrations of Zn (0.5, 5, 50, or 500 mg kg DW^-1^) as nanomaterial (NM) ZnO **(A,C)** or ionic Zn^2+^**(B,D)**.** Intake for each scenario is modeled for seven age-mass classes ranging from child to adult females and males. The dashed line represents the chronic oral RfD value for Zn. Data represent the mean and standard error (*n* = 4–5).

**FIGURE 2:**
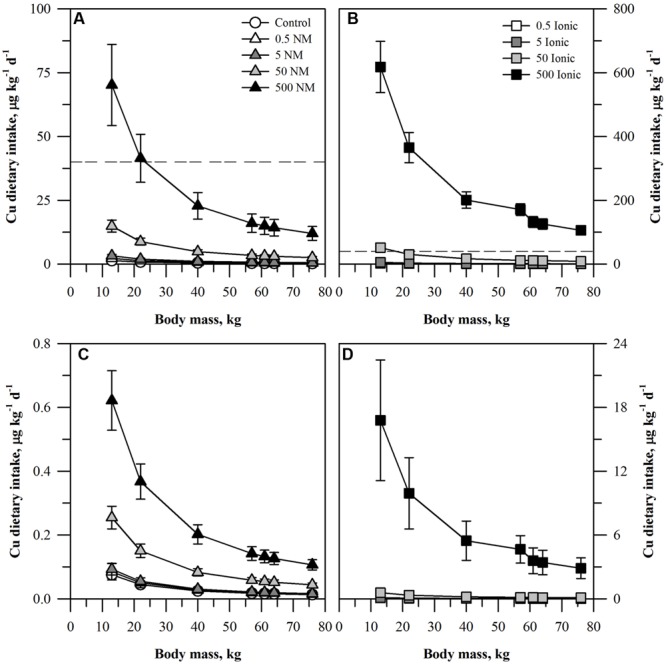
**Dietary intake models for Cu based on consumption of a single serving of unpeeled **(A,B)** or peeled carrot **(C,D)** grown in sand culture in the presence of four final concentrations of Cu (0.5, 5, 50, or 500 mg kg DW^-1^) as NM CuO **(A,C)** or ionic Cu^2+^**(B,D)**.** Intake for each scenario is modeled for seven age-mass classes ranging from child to adult females and males. The dashed line represents the chronic oral RfD value for Cu. Data represent the mean and standard error (*n* = 4–5).

**FIGURE 3:**
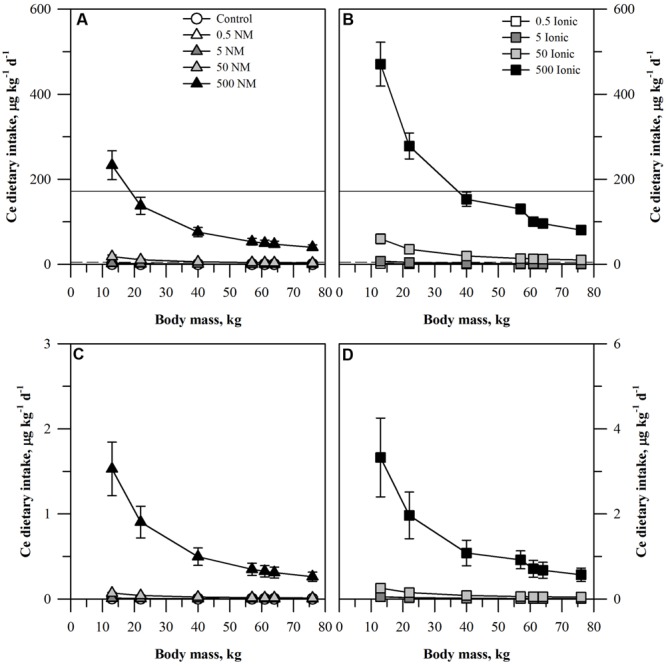
**Dietary intake models for Ce based on consumption of a single serving of unpeeled **(A,B)** or peeled carrot **(C,D)** grown in sand culture in the presence of four final concentrations of Ce (0.5, 5, 50, or 500 mg kg DW^-1^) as NM CeO_2_**(A,C)** or ionic Ce^4+^**(B,D)**.** Intake for each scenario is modeled for seven age-mass classes ranging from child to adult females and males. As there is no oral RfD for Ce, values for comparison were based on the mean (solid line) or median (dashed line) oral RfD calculated from nine other rare earth elements. Data represent the mean and standard error (*n* = 4–5).

The projected dietary intake of each element for the seven age-mass classes was estimated for a single serving of peeled or unpeeled carrots grown in the presence of the ENM or ionic form of the element (**Figures 1–3**). For unpeeled carrots (**Figures 1A,B**), the projected dietary intake for both forms for the two lower treatment concentrations and the control fell far below the oral RfD for Zn. The dietary intake from unpeeled carrots grown in the two highest concentrations of ionic Zn exceeded the oral RfD for some age-mass categories. For the 50 mg kg DW^-1^ ionic Zn treatment, the oral RfD was exceeded only for the smallest age-mass class (children 1–3 years) while for the 500 mg kg DW^-1^ ionic Zn treatment exceeded the oral RfD for all child age-mass classes from age 1 to 13 years. By comparison, the 50 mg kg DW^-1^ ENM Zn treatment fell below the Zn oral RfD and the 500 mg kg DW^-1^ ENM Zn treatment was similar to the corresponding ionic treatment. The projected dietary intake values calculated for the peeled carrots (**Figures 1C,D**) were >100-fold lower than the oral RfD and were considerably lower than the corresponding values for the unpeeled carrots. Projected dietary intake from peeled carrots treated with ionic Zn were consistently higher than the corresponding carrots from the ENM treatment.

The dietary intake results for the two lower concentrations of Cu (**Figure 2**) fell below the Cu oral RfD for both the ENM and ionic Cu treatments. The oral RfD was reached for unpeeled carrots (**Figures 2A,B**) only for the 500 mg kg DW^-1^ ENM Cu treatment for the two smallest age-mass classes. The values were higher for the two largest ionic treatments. The projected dietary intake values for the 50 mg kg DW^-1^ ionic Cu treatment exceeded the oral RfD for the smallest age-mass classes. All treatments for the 500 mg kg DW^-1^ ionic Cu treatment exceeded the oral RfD for Cu, a pattern much different from the corresponding CuO treatment. As with the peeled carrots and Zn, the projected dietary intake of Cu treated, peeled carrots (**Figures 2C,D**) fell ∼100-fold below the Cu oral RfD for all of the ENM treatments. The same was true for most of the ionic Cu treatments, except that the values were ∼10-fold higher than the ENM treatments with the exception of the highest ionic Cu treatment noted above.

The projected dietary intake values from the carrot receiving the ionic Ce treatment was generally double that from the plants receiving CeO_2_ ENMs for each treatment concentration and for each age-mass class. Interpretation of the results with respect to the oral RfD depended on which surrogate value for the Ce oral RfD was considered. If the results are compared to the mean oral RfD calculated for the nine other rare earth elements, then the only treatments where the mean oral RfD were exceeded were for the highest concentration of the ionic Ce treatment and the two smallest age-mass classes and also for the highest concentration of the CeO_2_ treatment and the smallest age-mass class. In contrast, if the median value was used, then the median oral RfD would be exceeded for the ionic treatment for all age-mass classes for the 50 and 500 mg kg DW^-1^ ionic treatments and the two smallest age-mass classes for the 5 mg kg DW^-1^ ionic treatment. For the CeO_2_ treatment, the value for all age-mass classes from the 500 mg kg DW^-1^ treatment and the two smallest age-mass classes from the 50 mg kg DW^-1^ ionic treatments would exceed the median oral RfD.

### Reverse Projections of Dietary Intakes of Zn or Cu

The forward modeling of dietary intakes was based on the consumption of a single serving of carrot on a single day. The reverse projections were conducted to provide an additional perspective to relate accumulation of Zn, Cu, or Ce in carrot to dietary intake. Solving the dietary intake equation for fresh weight tissue concentration provided values of the concentrations in the carrot tissue required to reach the oral RfD in a single serving in a single day. The values for Zn ranged from approximately 63 to 368 mg kg FW^-1^ for Zn and from approximately 8 to 49 mg kg FW^-1^ for Cu (**Table 1**). The values for Ce depend on the estimated oral RfD used. If the more conservative median oral RfD is used, the values range from 1 to >6 mg kg FW^-1^. Using the mean oral RfD for the nine other REEs, the range is higher, from 36 to >210 mg kg FW^-1^. By comparison, the maximum fresh weight Zn concentration observed for a single replicate of any ENM or ionic Zn treatments was 180.5 or 173.0 mg Zn kg FW^-1^, respectively. For Cu the values were 27.7 and 174.8 mg Cu kg FW^-1^, respectively. These values for ENM Zn fall between the upper two child age-mass classes (4–8 years and 9–13 years) and for ENM Cu fall between the upper child age-mass class (9–13 years) and the adolescent class (14–18 years). The value for ionic Zn is in the same age-mass range as ENM Zn but for ionic Cu, is at least threefold greater than the concentration of the largest age-mass class (males, 19–30 years). Obviously the highest fresh weight Ce concentrations observed for both ENM- and ionic-treated plants exceeded the values calculated for Ce based on the lower median oral RfD. When compared to the values based on the mean oral RfD, the maximum fresh weight concentrations observed corresponded to the lower three age-mass classes. Additional efforts from the reverse projections, expressing the results in terms of either the number of servings of carrot or the mass of carrot consumed to exceed the oral RfD, are presented in the Supplementary Material.

**Table 1:** Calculated tissue fresh weight concentrations of Zn, Cu, or Ce obtained from the reverse modeling efforts, that would be necessary to reach the oral RfD value in a single serving of standard mass for a given body mass of the individual consuming the carrot.

		Tissue fresh weight concentration (mg kg FW^-1^) necessary to reach oral RfD in one serving			
Age-mass class	Body mass, kg	Zn	Cu	Ce (Mean oral RfD)	Ce (Median oral RfD)
1–3	13	62.9	8.4	36.0	1.0
4–8	22	106.5	14.2	60.9	1.3
9–13	40	193.6	25.8	110.6	2.8
14–18, F	57	275.8	36.8	157.7	4.6
19–30, F	61	295.2	39.4	168.7	4.9
14–18, M	64	309.7	41.3	177.0	5.2
19–30, M	76	367.7	49.0	210.2	6.1

Maximum observed ENM FW conc.		180.5	27.7	65.8	
Maximum observed ionic FW conc.		173.0	174.8	126.9	

*For Ce, the data are calculated for both the mean and median oral RfD derived from nine other rare earth elements. For comparison, the maximum values for fresh weight tissue metal concentration from engineered nanomaterial (ENM) or ionic treated plants are shown.*

## Discussion

The most likely route by which NMs could be introduced into agricultural systems is through land application of biosolids (Benn and Westerhoff, 2008; Limbach et al., 2008; Mueller and Nowack, 2008; Gottschalk et al., 2009; Zheng et al., 2011; Priester et al., 2012). Biosolids are known to introduce metals into agricultural systems and Ceiling Concentration Limits (CCL) have been established in USEPA 40 CFR 503.13 for several metals and metalloids. For example, the CCL for Zn and Cu are 7,500 and 4,300 mg kg DW^-1^, respectively. Tangible data on the contribution of ENMs to the metal concentration in biosolids is lacking but there have been some estimates (Mueller and Nowack, 2008; Gottschalk et al., 2009; Tourinho et al., 2012). The estimated concentrations for Zn and Cu fall below the CCL values above. Other metals associated with ENMs, such as Ag or Ti are considered by Part 503.13. Additionally, formulations of ZnO, CuO, or CeO_2_ are being investigated as a strategy to enhance essential or beneficial nutrients for crop plants (DeRosa, 2010; Dimkpa et al., 2012a), while other ENMs are being included in nano-enabled agricultural products (Gogos et al., 2012; Mura et al., 2013; Suppan, 2013). With multiple possible routes of ENM entry into soil, the dissolution of the metallic oxide ENMs could increase the concentration of metals in those systems. In the absence of information on realistic environmental concentrations of ENMs (or metals from ENMs) a common practice in experimental studies has been to include a wide range of ENM concentrations in experiments, spanning multiple orders of magnitude. The inclusion of comparable ionic treatments has also been employed to provide a basis of comparison for the ENM treatments. Together these approaches provide a broad perspective from which to consider potential impacts, including potential worst case scenarios should highly elevated concentrations of metals from ENMs occur in environmental or agricultural systems.

There were two principal objectives of the research here. The first was to demonstrate the degree to which accumulation of metals from three metallic oxide ENMs (ZnO, CuO, and CeO_2_) in carrot might contribute to the dietary intake of the component metals and to relate those projections to an oral RfD for chronic toxicity. The results for Zn or Cu indicate that the oral RfD would be exceeded only under highly unrealistic, worst-case scenarios, such as growth in the presence of much higher concentrations of ZnO or CuO than are reasonably expected to occur or daily consumption of excessive amounts of unpeeled carrots. In terms of the second objective, peeling was projected to decrease substantially the dietary intake of all metals. In all cases, the projected dietary intake of these two metals were lower than the ionic treatments in nearly all cases, suggesting that for carrot and in terms of the total metal concentration that the ENMs ZnO and CuO do not necessarily present a greater risk to food safety. Whether this is true for other crop plants, including other root or tuberous vegetables or other metallic oxide ENMs, would need to be determined through additional studies.

The lack of an established oral RfD for Ce makes interpretation of the projected dietary intakes of Ce difficult. The mean and median values for the nine REEs were used here only to provide two potential perspectives. Cerium has been a component of REE fertilizers for decades and there are several studies reporting the concentrations of Ce that result in soils and crop tissues (Wen et al., 2001; Tyler, 2004; Brioschi et al., 2012; Silvia et al., 2013), yet these results have not prompted agencies to establish an oral RfD for this element. However, two recent studies examining the trophic transfer of Ce from ENMs through simulated food chains have offered data that may accentuate the need for guidelines for Ce in agricultural plants (Hawthorne et al., 2014; Majumdar et al., 2015). Studies with zucchini (*Cucurbita pepo*) and kidney bean (*Phaseolus vulgaris*) each showed that when plants were exposed to CeO_2_ ENMs or bulk CeO_2_, that accumulation of Ce was greater for the ENM form. In addition, crickets (*Acheta domesticus*) consuming zucchini leaves or Mexican bean beetles (*Epilachna varivestis*) consuming bean leaves retained more Ce from ENM-treated plants than bulk CeO_2_-treated plants. Transfer of Ce from these herbivorous insects to invertebrate predators, wolf spiders (family Lycosidae) or spined soldier bugs (*Podisus maculiventris*), respectively, was also greater from herbivorous insects that consumed ENM-treated plants as compared to bulk CeO_2_. Such results imply that trophic transfer of Ce from agricultural plants to humans might also occur in scenarios where Ce from CeO_2_ ENMs is present. As the use of CeO_2_ ENMs in commercial products and industrial processes is rapidly expanding, there may now be a greater need to establish an oral RfD or comparable toxicological limit for Ce in foods.

Overall the dietary intake projections used here establish a foundation on which to conduct similar efforts with nearly any combination of nanomaterial and plant or processed food. The accumulation in other food plants would need to be studied separately to determine if there were any scenarios specific to a particular crop species that need to be considered. It would also be possible to aggregate data from multiple plant species grown in the presence of the same ENM to consider the implications of a complex diet on dietary exposure. Subsequent models could be made more robust with the inclusion of additional information such as the *in situ* chemical form of ENM (or metal) in the plant tissue, fate in the human body (e.g., gastrointestinal absorption, elimination), and nano-specific effects of the ENM. Discriminating between ionic and nano-effects is recognized as an important research need in the field of nanotoxicology (Notter et al., 2014). In the absence of data on specific nano-mediated effects, there is a natural tendency to compare the two chemical forms based on total metal as this at least provides an established frame of reference. One advantage to the strategy here is that it aligns with currently regulatory practice which assumes that ENM-mediated effects are comparable to ionic effect. Whether or not this contention is valid will require additional research, yet in the context here for carrot where the risk of dietary intake from the ENM-treated plants is no worse than the ionic-treated plants, the information returned offers some regulatory perspective on which to begin to evaluate risk. Simple projections like those used here have value in that they provide a means to frame the scope of risk associated with a potential combination of plant food and NMs and to set priorities for future research.

## Author Contributions

SE, SB, and PK conducted the preliminary experiments to generate the body of prior data used for the modeling in the current study. JW and XM worked with SE to evaluate the data and models presented here and relate the results to the toxicological endpoints. All authors contributed to the preparation of the manuscript.

## Conflict of Interest Statement

The authors declare that the research was conducted in the absence of any commercial or financial relationships that could be construed as a potential conflict of interest.

The handling Editor NM declares that, despite hosting the Research Topic “Nanotoxicology and environmental risk assessment of engineered nanomaterials (ENMs) in plants” together with co-author JW, the review process was handled objectively.
