# Supplementary material for: Projected Dietary Intake of Zinc, Copper, and Cerium from Consumption of Carrot (Daucus carota) Exposed to Metal Oxide Nanoparticles or Metal Ions
Source: Front Plant Sci. 2016 Feb 24;7:188. doi: 10.3389/fpls.2016.00188 (PMC4764699; doi:10.3389/fpls.2016.00188)
Supplement: Supplementary file 1 [file Data_Sheet_1.DOCX]

Supplementary information

**Methods**

Preparation of nanomaterial solutions and plant exposure conditions

Seeds of carrot (Daucus carota cv Danvers Half Long, Burpee Seeds and Plants (W. Atlee Burpee & Co, Warminster, PA) were planted in plastic pots with 1.3 kg of dry coarse sand wetted with deionized water to 80% of field capacity. The carrot seeds were germinated in a phytotron (16 h photoperiod, 200-350 μmol m^-2^ s^-1^, 18-22°C, ambient relative humidity) and thinned to one plant per pot after emergence. Plants were grown for 16 weeks and were watered weekly with 0.05 L of a nutrient solution with 0.6 mM KNO_3_, 0.4 mM Ca(NO_3_)_2_, 0.05 mM NH_4_H_2_PO_4_, 0.1 mM MgSO_4_, 50 μM KCl, 12.5 μM H_3_BO_3_, 1 μM MnSO_4_, 1 μM ZnSO_4_, 0.5 μM CuSO_4_, 0.1 μM NiSO_4_, and 0.1 μM H_2_MoO_4_. The solution was buffered with 1 mM n-morpholinoethanesulfonic acid (MES), titrated to pH 6.0 with KOH. Iron was provided as 10 μM Fe-EDTA. Pots were watered with deionized water as needed to maintain 80% of field capacity.

The three ENMs used were obtained from US Research Nanomaterials, Inc. (Houston, TX) and were prepared as described in Ebbs et al. (2016). The ENMs were ZnO (30-40 nm, aqueous solution), CuO (25-55 nm, powder form), and CeO_2_ (30-50 nm, aqueous solution). Nanomaterial stock solutions containing ZnO or CuO were prepared to provide final concentrations of Zn or Cu at 1, 10, 100, or 1,000 mg L^-1^. The ZnO or CeO_2_ solutions were prepared by diluting the required volume of the commercial ENM solution with18 mΩ deionized water and sonicated (130 W, 20 kHz, model VCX 130, Sonics & Materials Inc., Newtown, CT) for 15 min. For each CuO solution, the necessary mass of the nanopowder was mixed with deionized water and sonicated as described above. A Zetasizer Nano ZS90 (Malvern Instruments, UK) was used to determine the hydrodynamic size for each of the nanomaterial suspensions. In parallel, stock solutions of the sulfate salts of ionic Zn, Cu, or Ce (Fisher Scientific (New Jersey, USA) were prepared at the same four concentrations of total Zn, Cu, or Ce.

The experimental design from this prior study consisted of either the nanomaterial or corresponding ion solution at one the four indicated concentrations (i.e., eight treatments total) and a control treatment (i.e., no metal). Each treatment was replicated five times. The Zn, Cu, and Ce treatments were not simultaneous but were conducted on three different sets of carrot plants. The pots were treated once per week for 13 weeks with 0.05 L of either one of the ENM or ionic stock solutions. By the end of the 13 week treatment period, the final concentration of Zn, Cu, or Ce in the pots was 0.5, 5, 50, or 500 mg kg DW^-1^. During the 13 week exposure period, nutrient solution and deionized water were added to the pots weekly. Plants were given their respective treatment once per week for 13 weeks and were harvested.

At harvest, plants were removed from the pots, separated into shoots and roots and then rinsed with deionized water. The carrot taproot was gently abraded with a brush to insure removal of any adhering sand particles. The taproot was peeled with a standard vegetable peeler, removing the outer 2-3 mm of the carrot surface (i.e., the “peels”). The fresh weight of the tissues was determined and the tissues were then dried to constant mass at 60°C. The percent water content of the taproot tissues was calculated based on the fresh and dry weight values. The dried tissues were ground to a particle size of <5 mm and digested using EPA method 3050b using a combination of trace metal grade nitric acid and 30% hydrogen peroxide (USEPA 1996). The digested samples were analyzed for the metal using inductively coupled plasma mass spectroscopy (ICP-MS, Agilent 7500ce, Santa Clara, CA). The dry weight concentration data for the carrot tissue and percent water content of each tissue were used to determine the fresh weight concentration of Zn, Cu, or Ce. The dietary intake projections were based on consumption of fresh (i.e. uncooked) carrot tissue so fresh weight tissue concentrations were needed for the models.

Data analysis

The taproot fresh weight concentration was subjected to a three-way ANOVA with treatment concentration, chemical form (ENM or ionic), and root tissue (peel or flesh) as the main effects. Post hoc analysis utilized Duncan’s test. Where interactions between the main effects were present, the data was analyzed again using the interactions means for each pair of main effects, or the interaction mean for all three factors, and a one-way ANOVA, thereby treating each combination of main effects as an individual treatment. As the plants used for the Zn and Cu treatments were grown sequentially not simultaneously during the course of this research, data were not compared between elements but only within each element.

**Results**

Accumulation of Zn, Cu, or Ce from ENM or ionic treatments

For tissue fresh weight Zn concentration there was no significant effect of chemical form on tissue Zn concentration (Table S1) yet there was a significant interaction between concentration and peeling treatment. Tissue Zn concentrations generally increased with increasing Zn treatment concentration for unpeeled carrots (but not peeled carrots) and were higher for each treatment for unpeeled carrot taproots than for peeled carrot taproots. This pattern was interpreted as evidence that the Zn from the ionic treatment penetrated into the carrot taproot interior more readily that Zn from the ZnO treatment.

There was a three-way interaction between the main effects for Cu (Table S1-S2). Unlike the Zn treatments, there was a difference in accumulation of Cu from the ionic treatment as compared to the ENM CuO treatment. There were no significant differences amongst the control and the lower two treatment concentrations (0.5 and 5 mg kg DW^-1^) but differences were significant amongst the higher two concentrations (50 and 500 mg kg DW^-1^) and for the unpeeled carrots. Accumulation of Cu from the ionic treatment was consistently higher for the ionic Cu treatment as compared to the CuO treatment. As for Zn, the majority of Cu was retained in the peel, hence the peeling treatment resulted in a significant reduction in Cu concentration in the edible taproot flesh. Results very similar to the pattern for Cu were obtained for Ce where a three-way interaction was also observed.

Additional results from reverse projections

Additional reverse projections were conducted to solve for the number of servings or mass of unpeeled or peeled carrot to be consumed to reach the oral RfD. The values obtained are quite large, much larger than would typically be consumed on a typical day and even larger for peeled carrots as compared to unpeeled carrots (Figures S1-S6). The first aspect of the reverse modeling solved for the number of servings of an unpeeled or peeled carrot needed to reach the oral RfD in a single day (Figure S1-S3). Four trends are evident in these results for both metals. The first and most evident trend is that not surprisingly, the number of servings necessary increases with decreasing metal treatment concentration and increasing body mass. Second, the number of servings needed to reach the oral RfD for either element ranged widely depending on the form of Zn, Cu, or Ce the plants received and whether the calculations were based on unpeeled or peeled carrots. Servings from approximately <20 to >3,600 per day were calculated for Zn (Figure S1) and for Cu (Figure S2). The two lower and two higher treatment concentrations of either Zn or Cu were generally grouped together in the relationships. For Ce, the results were determined for both the mean and median oral RfD for the nine rare earth. For the median oral RfD (5 μg kg^-1^ d^-1^), the two higher treatment concentrations also grouped together but there was considerable variability in the values for the two lower concentrations (Figure S3). The range in the number of servings was also much wider, ranging from <20 to > 7,000 and proportionally higher if the calculated were based on the mean oral RfD of 171.5 5 μg kg^-1^ d^-1^ (data not shown). Finally, the number of servings was lower for carrots treated with ionic Zn, Cu, or Ce compared to the nanomaterial form and at least an order of magnitude higher for unpeeled carrots as compared to peeled carrots. Thus unpeeled, ionic-treated carrots would require the fewest number of servings to reach the oral RfD and peeled, ENM-treated carrots generally the most.

The same fundamental trends were evident if the dietary intake equation was solved for the mass of carrot consumed and the results expressed in terms of the amount carrot per day (in kg) necessary to reach the oral RfD in a single day (Figures S4-S6). The mass values obtained were quite large, corresponding in many cases to tissue concentrations well below the oral Rfd values. For example irrespective of the metal considered, for adolescent and adult age-mass classes, calculations indicated that from approximately 2 to 15 kg of unpeeled carrots would be required in a day to reach the oral RfD and from >10 to >200 kg of peeled carrots if either Zn or Cu are considered (Figures S4-S5). The values for Ce (Figure S6) based on the median oral RfD are much smaller for unpeeled carrots (<1 to >4.5 kg) and much larger for peeled carrots (<1.5 to >380). Even larger values for Ce would be projected if the results were based on the mean oral RfD (data not shown). The calculated values for the child age-mass classes are understandably smaller, but were no smaller than 0.35 to 0.4 kg of either unpeeled or peeled carrots per day, and those values were observed at the highest treatment concentrations. These trends are generally the same for all three elements. The mass to be consumed was also consistently larger for the ENM-treated plants, corresponding to the lower tissue concentration generally observed.

**References**

Ebbs, S.D., Bradfield, S., Kumar, P., Musante, C., White, J.C. Ma, X. (2016). Accumulation of zinc, copper, or cerium in carrot (Daucus carota) exposed to metal oxide nanoparticles and metal ions. Environ. Sci.: Nano. In press.

USEPA (1996). "Method 3050b: Acid Digestion of Sediments, Sludges, and Soils " in Test Methods for Evaluating Solid Waste Physical/Chemical Methods, SW-846 ed. (Washington, DC: Environmental Protection Agency, Office of Solid Waste), 12 pp.

**Table S1.** Fresh weight Zn, Cu, or Ce concentrations in unpeeled or peeled mature carrot taproots for plants grown in sand culture treated with nanomaterial ZnO, CuO, CeO_2_ or ionic Zn^2+^, Cu^2+^, Ce^4+^ at concentrations ranging from 0.5 to 500 mg kg DW^-1^. The letters in parentheses indicate the results of a one-way ANOVA conducted on the interaction means for zinc (concentration x peeling) or for Cu and Ce (concentration x form x peeling). For Zn, form had no significant effect so the concentrations for each peeling treatment were considered separately. The results for Cu and Ce showed a three-way interaction so each combination of main effects was expressed as a unique treatment.

|  | **Concentration of Zn, Cu, or Ce (mg kg FW^-1^) in unpeeled or peeled carrots** | | | | | | | | | | | | | | |
| --- | --- | --- | --- | --- | --- | --- | --- | --- | --- | --- | --- | --- | --- | --- | --- |
| **Treatment,**  **mg kg DW^-1^** | **Zinc** | | | | |  | **Copper** | | | |  | **Cerium** | | | |
| *Unpeeled* | Nanomaterial | | Ionic | | |  | Nanomaterial | | Ionic | |  | Nanomaterial | | Ionic | |
| 0 | 2.3 | +0.3 | 2.3 | +0.3 | (a) |  | 0.3 | +0.02(a) | 0.3 | +0.02(a) |  | 0.1 | +0.004(a) | 0.1 | +0.004(a) |
| 0.5 | 1.7 | +0.4 | 4.6 | +1.0 | (a) |  | 0.5 | +0.01(a) | 0.5 | +0.06(a) |  | 0.4 | +0.2(ab) | 0.3 | +0.1(ab) |
| 5 | 2.8 | +0.4 | 5.8 | +1.4 | (a) |  | 0.7 | +0.01(a) | 1.1 | +0.1(a) |  | 0.6 | +0.2(b) | 1.6 | +0.2(c) |
| 50 | 66.4 | +1.3 | 36.7 | +6.2 | (a) |  | 3.1 | +0.02(ab) | 10.8 | +1.4(ab) |  | 3.8 | +0.8(cd) | 12.5 | +2.4(d) |
| 500 | 103.8 | +30.9 | 94.3 | +27.0 | (b) |  | 14.7 | +0.03(b) | 129.5 | +16.8(c) |  | 48.8 | +7.1(e) | 98.7 | +10.8(f) |
| *Peeled* | Nanomaterial | | Ionic | | |  | Nanomaterial | | Ionic | |  | Nanomaterial | | Ionic | |
| 0 | 0.2 | +0.02 | 0.2 | +0.02 | (a) |  | 0.02 | +0.002(a) | 0.02 | +0.002(a) |  | 0.001 | +0.0002(a) | 0.001 | +0.0002(a) |
| 0.5 | 0.1 | +0.01 | 0.2 | +0.01 | (a) |  | 0.02 | +0.005(a) | 0.02 | +0.001(a) |  | 0.001 | +0.0004(a) | 0.01 | +0.002(a) |
| 5 | 0.1 | +0.01 | 0.3 | +0.02 | (a) |  | 0.02 | +0.004(a) | 0.02 | +0.001(a) |  | 0.002 | +0.001(a) | 0.01 | +0.001(a) |
| 50 | 0.2 | +0.02 | 3.9 | +0.9 | (a) |  | 0.05 | +0.007(a) | 0.1 | +0.02(a) |  | 0.01 | +0.001(a) | 0.1 | +0.01(a) |
| 500 | 0.2 | +0.03 | 4.9 | +0.9 | (a) |  | 0.3 | +0.02(a) | 3.5 | +1.2(ab) |  | 0.3 | +0.1(ab) | 0.7 | +0.2(b) |

**Table S2.** The *p*-values resulting from the three-way ANOVA examining the effects of concentration, form (nanomaterial or ionic), or peeling (unpeeled or peeled) on fresh weight concentration of zinc, copper, or cerium in carrot taproots.

| **Main Effect** | **Zinc** |  | **Copper** |  | **Cerium** |
| --- | --- | --- | --- | --- | --- |
| Concentration (C) | <0.001 |  | <0.001 |  | <0.001 |
| Form (F) | n.s. |  | <0.001 |  | <0.001 |
| Peeling (P) | <0.001 |  | <0.001 |  | <0.001 |
| C x F | n.s. |  | <0.001 |  | <0.001 |
| C x P | <0.001 |  | <0.001 |  | <0.001 |
| F x P | n.s. |  | <0.001 |  | <0.001 |
| C x F x P | n.s. |  | <0.001 |  | <0.001 |

**Supplementary Figure Legends**

**Figure S1.** Results of the reverse modeling to estimate the number of servings of a treated carrot with a known Zn concentration that would be required to reach the oral RfD value for Zn (300 μg kg^-1^ d^-1^) given the body mass of the individual and a standard mass per serving of carrot. Data are derived from carrots grown in sand culture in the presence of 0.5, 5, 50, or 500 mg Zn kg DW^-1^ nanomaterial (NM) ZnO or ionic Zn^2+^. Data represent the mean and standard error (*n*=4-5).

**Figure S2.** Results of the reverse modeling to estimate the number of servings of a treated carrot with a known Cu concentration that would be required to reach the oral RfD value for Cu (40 μg kg^-1^ d^-1^) given the body mass of the individual and a standard mass per serving of carrot. Data are derived from carrots grown in sand culture in the presence of 0.5, 5, 50, or 500 mg Cu kg DW^-1^ nanomaterial (NM) CuO or ionic Cu^2+^. Data represent the mean and standard error (*n*=4-5).

**Figure S3.** Results of the reverse modeling to estimate the number of servings of a treated carrot with a known Ce concentration that would be required to reach the median oral RfD value calculated for nine rare earth elements (5 μg kg^-1^ d^-1^) given the body mass of the individual and a standard mass per serving of carrot. Data are derived from carrots grown in sand culture in the presence of 0.5, 5, 50, or 500 mg Ce kg DW^-1^ nanomaterial (NM) CeO_2_ or ionic Ce^4+^. Data represent the mean and standard error (*n*=4-5).

**Figure S4.** Results of the reverse modeling to estimate the mass of a treated carrot with a known Zn concentration that would be required to reach the oral RfD value for Zn (300 μg kg^-1^ d^-1^) given the body mass of the individual and a single serving of carrot. Data are derived from carrots grown in sand culture in the presence of 0.5, 5, 50, or 500 mg Zn kg DW^-1^ nanomaterial (NM) ZnO or ionic Zn^2+^. Data represent the mean and standard error (*n*=4-5).

**Figure S5.** Results of the reverse modeling to estimate the mass of a treated carrot with a known Zn concentration that would be required to reach the oral RfD value for Cu (40 μg kg^-1^ d^-1^) given the body mass of the individual and a single serving of carrot. Data are derived from carrots grown in sand culture in the presence of 0.5, 5, 50, or 500 mg Cu kg DW^-1^ nanomaterial (NM) CuO or ionic Ce^2+^. Data represent the mean and standard error (*n*=4-5).

**Figure S6.** Results of the reverse modeling to estimate the mass of a treated carrot with a known Zn concentration that would be required the median oral RfD value calculated for nine rare earth elements (5 μg kg^-1^ d^-1^) given the body mass of the individual and a single serving of carrot. Data are derived from carrots grown in sand culture in the presence of 0.5, 5, 50, or 500 mg Ce kg DW^-1^ nanomaterial (NM) CeO_2_ or ionic Ce^4+^. Data represent the mean and standard error (*n*=4-5).

Figure S1

Figure S2

Figure S3

Figure S4

Figure S5

Figure S6
